# Tissue-aggressive inflammatory response defines the tissue aggressiveness of the post-infarction milieu

**DOI:** 10.1186/cc13381

**Published:** 2014-03-17

**Authors:** E Grins, L Algotsson, H Engblom, M Carlsson, H Arheden, S Jovinge

**Affiliations:** 1 Scania University Hospital, Lund University, Lund, Sweden

## Introduction

A potential measure of post-ischemic milieu in myocardium subject to acute ischemic injury is to assess the so- called myocardial salvage index (SI) by relating the final infarct size to the initial myocardium at risk (MaR). Cardiac magnetic resonance (CMR) has previously been shown to enable determination of both infarct size using late gadolinium enhancement and MaR using T2- weighted imaging. This technique could thus potentially be used to identify inflammatory responses that could be targeted to accomplish cardioprotection. The aim of this study was to relate SI, as determined by CMR, to the inflammatory response in patients with acute myocardial infarction.

## Methods

Fifteen patients with first-time ST-elevation myocardial infarction were included in the study. All patients underwent primary PCI due to an acute occlusion in one branch of the left coronary artery. Final infarct size and MaR was determined by CMR performed 1 week after the acute event. The ischemic time was defined as the time from pain onset to opening of the occluded vessel. Blood samples were taken for assessment of inflammatory response. Inflammatory cells were analyzed by flow cytometry in a BD FACS Aria. Parameters were gated against control antibody and fluorescence minus one strategy. Cytokine patterns were analyzed by BioRad BioPlex multiplex protein analysis technology.

## Results

The SI did not correlate with MaR (*P *= 0.2720, *R*^2 ^= 0.09191). The population was divided into the lowest half of the SI (representing the most hostile milieu; SI: 23 to 57%) and the upper half of the SI (representing the friendliest post-infarction milieu; SI: 71 to 95%). The patients' profile of adaptive inflammatory response was characterized by flow cytometry. The two groups did not differ with regard to their T-regulatory response (CD25+FoxP3+, *P *= 0.7203) or NK-cell (CD3CD56+,

*P *= 0.5742) response. However, the proportion of TH1 (CD4^+ ^IFNγ^+^) cells was higher in the group with the lowest SI (57.47 ± 4.805 vs. 38.10 ± 6.514, *P *= 0.0349).

## Conclusion

This is the first study that identifies inflammatory cell patterns which influence the hostility of the infarction milieu *in vivo. *The study indicates that SI can be used as an evaluator of post- ischemic milieu and that TH1 response is associated with unfavorable post-infarction injury and could therefore be a possible target for future studies to limit infarction size. The current study is, by its size and observational character, important; an argument for rather than a substitute for future interventional studies in this area.

